# Positive Association of Video Game Playing with Left Frontal Cortical Thickness in Adolescents

**DOI:** 10.1371/journal.pone.0091506

**Published:** 2014-03-14

**Authors:** Simone Kühn, Robert Lorenz, Tobias Banaschewski, Gareth J. Barker, Christian Büchel, Patricia J. Conrod, Herta Flor, Hugh Garavan, Bernd Ittermann, Eva Loth, Karl Mann, Frauke Nees, Eric Artiges, Tomas Paus, Marcella Rietschel, Michael N. Smolka, Andreas Ströhle, Bernadetta Walaszek, Gunter Schumann, Andreas Heinz, Jürgen Gallinat, The IMAGEN Consortium

**Affiliations:** 1 Ghent University, Faculty of Psychology and Educational Sciences, Department of Experimental Psychology and Ghent Institute for Functional and Metabolic Imaging, Ghent, Belgium; 2 Clinic for Psychiatry and Psychotherapy, Charité University Medicine, St Hedwig Krankenhaus, Campus Mitte, Berlin, Germany; 3 Physikalisch-Technische Bundesanstalt (PTB), Berlin and Braunschweig, Germany; 4 Department of Child and Adolescent Psychiatry, Central Institute of Mental Health, Mannheim, Germany; 5 Institute of Psychiatry, King's College London, London, United Kingdom; 6 Universitaetsklinikum Hamburg Eppendorf, Hamburg, Germany; 7 Department of Cognitive and Clinical Neuroscience, Central Institute of Mental Health, Mannheim, Germany; 8 Institute of Neuroscience, Trinity College Dublin, Dublin, Ireland; 9 Department of Addictive Behaviour and Addiction Medicine, Central Institute of Mental Health, Mannheim, Germany; 10 Institut National de la Santé et de la Recherche Médicale, INSERM Unit 1000 “Imaging & Psychiatry,” University Paris Sud, Orsay, France; 11 AP-HP Department of Adolescent Psychopathology and Medicine, Maison de Solenn, University Paris Descartes, Paris, France; 12 Rotman Research Institute, University of Toronto, Toronto, Canada; 13 Montreal Neurological Institute, McGill University, Montreal, Canada; 14 School of Psychology, University of Nottingham, Nottingham, United Kingdom; 15 Department of Genetic Epidemiology in Psychiatry, Central Institute of Mental Health, Mannheim, Germany; 16 Department of Psychiatry and Psychotherapy, Technische Universität Dresden, Dresden, Germany; 17 Neuroimaging Center, Department of Psychology, Technische Universität Dresden, Dresden, Germany; 18 Department of Psychiatry and Psychotherapy, University Medical Center Hamburg-Eppendorf, Hamburg, Germany; George Mason University/Krasnow Institute for Advanced Study, United States of America

## Abstract

Playing video games is a common recreational activity of adolescents. Recent research associated frequent video game playing with improvements in cognitive functions. Improvements in cognition have been related to grey matter changes in prefrontal cortex. However, a fine-grained analysis of human brain structure in relation to video gaming is lacking. In magnetic resonance imaging scans of 152 14-year old adolescents, FreeSurfer was used to estimate cortical thickness. Cortical thickness across the whole cortical surface was correlated with self-reported duration of video gaming (hours per week). A robust positive association between cortical thickness and video gaming duration was observed in left dorsolateral prefrontal cortex (DLPFC) and left frontal eye fields (FEFs). No regions showed cortical thinning in association with video gaming frequency. DLPFC is the core correlate of executive control and strategic planning which in turn are essential cognitive domains for successful video gaming. The FEFs are a key region involved in visuo-motor integration important for programming and execution of eye movements and allocation of visuo-spatial attention, processes engaged extensively in video games. The results may represent the biological basis of previously reported cognitive improvements due to video game play. Whether or not these results represent a-priori characteristics or consequences of video gaming should be studied in future longitudinal investigations.

## Introduction

The rapid growth of video game popularity in adolescents has generated concern among practitioners, parents, scholars and politicians. For violent video games, detrimental effects have been reported in social domains, namely increases in aggression and reductions of empathy and prosocial behaviour [1], [2]. But favourable effects of frequent video game playing have also been observed. It has been shown that action video game playing can enhance probabilistic inferences [3], as well as visual skills related to attention, memory and the spatial resolution of vision [4]–[7]. Furthermore, improvements in higher-level cognitive functions such as task switching, working memory and reasoning have been associated with improvements in a strategic video game [8]. Additionally, video games have been shown to enhance spatial skills [9] and motor skills, such as endoscopic surgical performance [10], [11].

Brain mapping studies have established that extensive experience with certain skills can alter brain activity during performance of that skill [12], [13] and enlarge brain structures typically engaged by a given activity [14]. Variations in brain structure have been associated with a broad spectrum of skills such as taxi driving [15], juggling [16], studying for medical exams [17], keyboard typing [18], morse-code [19] and musical skills [20].

Although behavioural studies have demonstrated effects on visual and cognitive skills, research on the structural correlates of frequent video game playing is still scarce. Of note is a study by Lövden et al. [21], in which healthy younger and older men performed a cognitively demanding computer game that required spatial navigation within a virtual environment while walking on a treadmill every other day over a period of 4 months. Structural images were acquired before training, after 4 months of training and 4 months after termination of training. The young and old experimental group had stable hippocampal volumes that were maintained 4 months after termination of training. In contrast, the young and old control group that walked on the treadmill but did not train with the spatial navigation task displayed volume decrements consistent with longitudinal estimates of age-related decline.

In a first structural study exploring the neural correlates of video game playing on the same data set as the present study we used voxel-based morphometry (VBM) to compare frequent (more than 9 h/week) with infrequent (less than 9 h/week) video game playing adolescents [22]. We found increased left striatal grey matter volume in frequent compared with infrequent video game players accompanied by stronger brain activity in left striatum during feedback of loss compared with no loss. Compared to VBM, the method employed previously [22], cortical thickness has been suggested to be a more sensitive parameter with a higher signal-to-noise ratio [23]-[26]. Furthermore cortical thickness has been shown to be associated with normal aging, cognitive performance and mental disorders.

To explore the association between spontaneous video game playing and cortical thickness, we analysed data from 152 14-year old adolescents from the IMAGEN project [27] including a questionnaire assessing video gaming frequency and high-resolution structural magnetic resonance imaging (MRI) scans.

## Materials and Methods

### Participants

152 healthy 14-year old adolescents (mean  = 14.4, SD = 0.03 years; 72 males, 80 females) were participants of the IMAGEN project, a European multi-centre genetic-neuroimaging study in adolescence [27]. Data from this project is stored on a data server operated according to European data protection law. The data access and overall scientific direction is regulated by a Project Executive Committee (PEC) chaired by the Scientific Co-ordinator (Gunter Schumann, IOP London). Written informed consent was obtained from all legal guardians and assent was obtained from the adolescents. All adolescents were recruited from secondary schools in Berlin. The study was approved by the ethics committee of the Medical Department of the University of Heidelberg. Participants with serious medical conditions such as brain tumours, neurological disorders like epilepsy or mental-health disorders were excluded. Mental health of all participants was assessed by means of self-rating and two external ratings (by their parents and a psychiatrist specialized in paediatrics) based on ICD-10 as well as DSM-IV (The Development and Well-Being Assessment Interview, DAWBA, [28]). None of the participants included in the present study received a psychiatric diagnosis.

### Scanning Sequence

For logistical reasons, structural images were collected on two scanners - a General Electric Signa Excite 3 T scanner (Milwaukee WI, USA) and a Siemens Verio 3 T (Erlangen, Germany). The participants scanned on the GE and Siemens scanners consisted, respectively, of 63 participants who played 14.4 hours per week on average (SD = 13.9) and 89 participants playing 11.4 hours per week on average (SD = 12.2); this difference was not significant (*t*(150) = 1.4, *p* = 0.16). The images were obtained using a three-dimensional T1-weighted magnetization prepared gradient-echo sequence (MPRAGE) based on the ADNI protocol (www.adni-info.org; GE scanner: repetition time  = 7.16 msec; echo time  = 3.02 msec; flip angle  = 8°; 256×256×166 matrix, 1.1×1.1×1.1 mm voxel size; Siemens scanner: repetition time  = 6.9 msec; echo time  = 2.93 msec; flip angle  = 9°; 240×256×160 matrix, 1.1×1.1×1.1 mm voxel size).

### Questionnaire

We administered a questionnaire assessing computer gaming behaviour (CSV-S, [29]) comprising of the questions: “How many hours do you play video games on average on a weekday?” and “How many hours do you play video games on average on a day during the weekend?”. Based on the hours indicated, we calculated the weekly hours spent playing video games. Moreover we categorized the participants according to the CSV-S score that defines excessive video game playing with a cut-off of a score of 4 and addiction with a cut-off of 7. This questionnaire was administered only in the Berlin sample of the IMAGEN study.

### Data Analysis

Cortical thickness was estimated from the structural magnetic resonance images using FreeSurfer software (http://surfer.nmr.mgh.harvard.edu/, [30]), a set of automated tools for reconstruction of brain cortical surface [31].

First, we used the T_1_-weighted images to segment white matter and to estimate the grey-white matter interface. This estimate of grey-white matter interface was used as the starting point of a deformable surface algorithm searching for the pial surface. The whole cerebral cortex of each participant was visually inspected for inaccuracies, only participants with no segmentation errors were included in the present data set (n = 6 additional subjects were excluded from further analysis, due to errors in segmentation at the transition between temporal lobe and insula). Local cortical thickness was estimated based on the difference between the position of equivalent vertices in the pial surface and grey-white matter interface. The surface of the grey-white matter border was inflated and differences between participants in the depth of gyri and sulci were normalized. Each participant's reconstructed brain was morphed and registered to an average spherical surface. In order to obtain difference maps of cortical thickness, the data were smoothed on the level of the sphere using a Gaussian smoothing kernel with a full-width half maximum of 15 mm. We used a multiple regression approach to explore the association between cortical thickness and self-reported average video gaming hours per week. To control for the effects of age, sex and scanner these variables were entered as additional regressors of no interest in the whole cortical surface multiple regression analysis. The resulting maps were initially thresholded with *p*<0.001. Then we performed Monte Carlo simulation cluster analysis in FreeSurfer qdec comprising the synthesis of white Gaussian noise on the estimated surface, smoothing and clustering to correct for multiple comparisons using a cluster threshold of *p*<0.01. Only clusters that survived this correction for multiple comparisons are reported.

## Results

On average the participants reported playing video games for an average of 12.6 (SD = 12.9, range = 63) hours during a typical week. There was a significant difference in hours of video game playing between males and females, with females playing less frequently (*t*(150) = 5.03, *p*<0.001). Participants indicating that they do not play video games during an average week were exclusively female. The group of participants reporting video game playing consisted of 54 females and 72 males. According to the CSV-S scoring schema 6 participants were classified as addicted and 13 as excessive users.

Cortical thickness differed between scanners (DLPFC: *t*(150) = 4.56, *p*<0.001; FEF: *t*(150) = 5.07, *p*<0.001); on the GE scanner cortical thickness values were higher compared to Siemens, therefore scanner was entered as a covariate of no interest in the main analysis.

**Figure 1:**
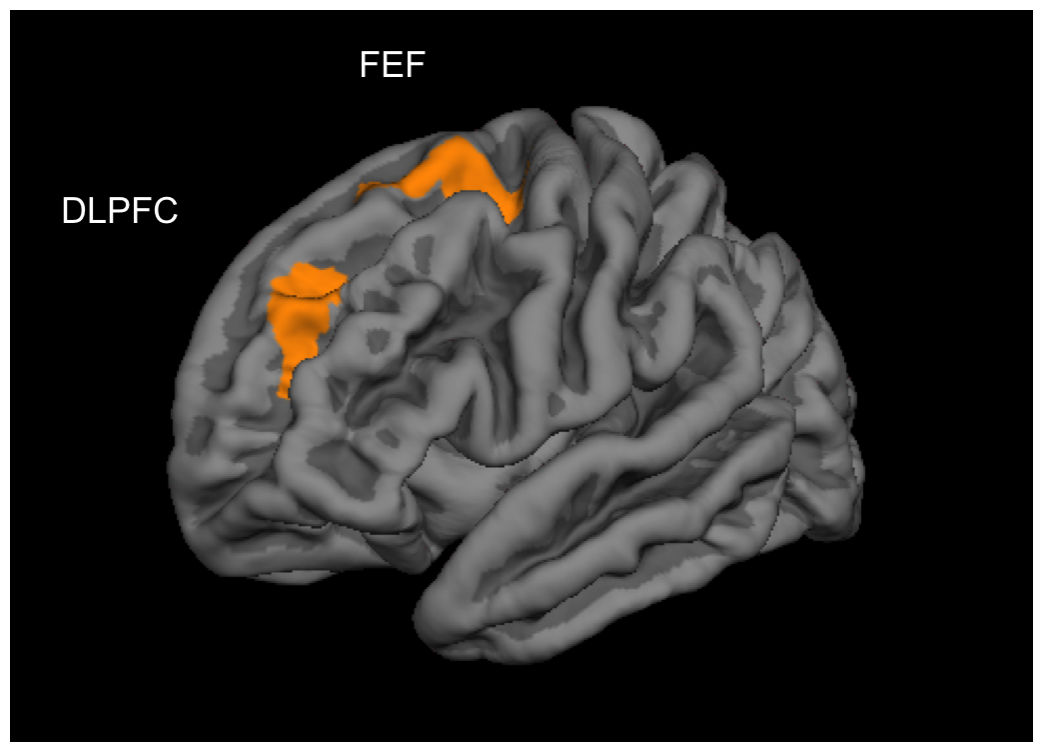
Significant clusters of the cortical thickness correlation with hours of video gaming per week in the left dorsolateral prefrontal cortex (DLPFC) and left frontal eye fields (FEF) (multiple comparison corrected, *p*<0.01).

**Figure 2:**
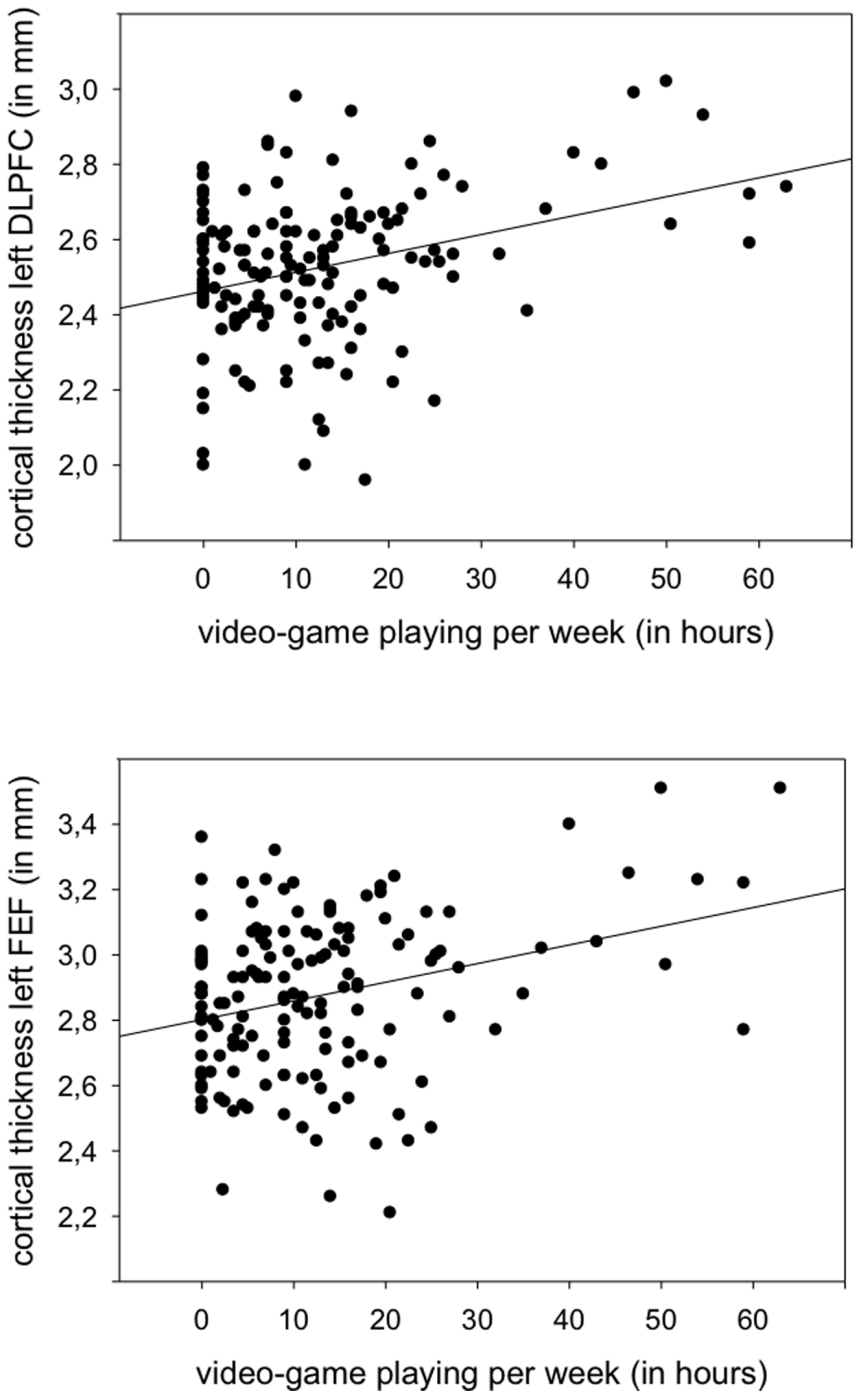
Scatter plot of the association between cortical thickness in left dorsolateral prefrontal cortex (DLPFC, top) and frontal eye fields (FEF, bottom) and hours of video gaming per week. Correlation coefficients are not reported since the brain regions are defined based on a whole brain analysis and applying statistics on these regions could be considered “double dipping” (Kriegeskorte et al., 2009).

When computing the main whole brain analysis to explore brain regions in which cortical thickness varied with hours per week of video gaming (controlling for age, sex and scanner), we found that the time playing video games correlated positively with cortical thickness in the left dorsolateral prefrontal cortex in left middle frontal gyrus extending into left superior frontal gyrus (DLPFC −40 40 24, according to [32]; BA 9 and 46, rostral middle frontal according to [33]; total surface area 710 mm^2^; total grey matter volume 2415 mm^3^) and left frontal eye fields (FEF, −19 −2 51; BA6, superior frontal area; total surface area 592 mm^3^; total grey matter volume 2089 mm^3^; [34], [35] (*Figure 1*). To illustrate the observed effects, and to exclude the possibility that outliers were driving the effects, a scatterplot is shown in *Figure 2*. There were no regions of significant negative correlation between cortical thickness and video game playing per week.

To explore the effects of the nuisance variables age, sex and scanner we computed the associations between these variables with cortical thickness extracted from the clusters in DLPFC and FEF. Age was only marginally associated with cortical thickness (DLPFC: *r*(152) = −0.123, *p* = 0.13; FEF: *r*(152) = −0.15, *p* = 0.065), whereas no significant difference was observed between female and male participants (DLPFC: *t*(150) = −0.32, *p* = 0.749; FEF: *t*(150) = −0.20, *p* = 0.839).

We compared cortical thickness in participants that were classified as excessive (n = 13) or addicted (n = 6) video gamers to the remaining participants in a post-hoc comparison. We found a significant difference in DLPFC (*F*(1,118) = 6.36, *p*<0.05; partial eta square: η^2^ = 0.051) when controlling for sex, age and scanner, but no significant effect in FEF (*F*(1,118) = 0.54, *p* = 0.464).

Socioeconomic background, approximated by means of the highest education of the parents, did not influence the association between video gaming hours per week and cortical thickness in DLFPC and FEF; when partialing out parental education the correlations were still significant (DLPFC: *r*(149) = 0.317, *p*<0.001; FEF: *r*(149) = 0.289, *p*<0.001).

Although one might be tempted to assume a positive association between body mass index (BMI) and video gaming, we did not find an association between self-reported video game hours at age 14 and BMI at age 16 (*r*(74) = −0.007, *p* = 0.95). However, we did not assess BMI at age 14 and can therefore not report results of concurrent video game behaviour and BMI.

## Discussion

Within the scope of the present study we investigated the structural correlates of video gaming frequency in a sample of 152 adolescents and found a positive correlation between self-reported hours of video gaming per week and cortical thickness in the left DLPFC and left FEF (also when controlling for sex, age, scanner and socioeconomic status). Reductions in cortical thickness associated with video gaming frequency were not observed.

Prefrontal cortex has been described as the substrate of executive control [36], [37]. DLPFC tends to be prominently involved in manipulating decision relevant information and during decisions involving conscious deliberation [38]. Extensive research has implicated DLPFC in working memory, a cognitive requirement for maintaining decision goals, considering options and integrating both to predict future outcomes [39]–[41]. Several studies indicate a general trend towards a lateralization in function of DLPFC: right DLPFC seems to be implicated when novel information requires responses that are drawn from memory, whereas left DLPFC is implicated when responses draw upon environmental evidence [42]. In a neuroimaging study on inductive inferences, the left DLPFC was more strongly involved during ambiguity resolution, in which explicit contextual cues guided decision-making, whereas right DLPFC was more prominently activated during memory-based retrieval of criterions for decisions [43]. In line with this prefrontal lateralization, studies on visual perceptual decision-making in monkeys [44] as well as humans [45] have reported activity in left DLPFC associated with the representation of differential output from neuronal populations representing different stimulus alternatives. The notion that the left DLPFC comprises a decision-making module is supported by the fact that its involvement is independent of the modality of perceptual input and independent of the response modality that participants use to communicate the outcome of their decision. The independence of left DLPFC activity from response modality has been demonstrated by comparing visual motion discrimination to which participants had to respond by means of button presses or saccadic eye movements [46]. Both conditions involved activation of left posterior DLPFC independent of the motor system that participants used to express their decision. The association between DLPFC and perceptual decision-making is further supported by a study on monkeys with lesions in the posterior DLPFC, which displayed impairments in discrimination tasks [47].

Interestingly, the FEFs have also been implicated in perceptual decision-making studies on monkeys [44] and humans [46]. Heekeren and colleagues [46] asked humans to make direction-of-motion judgements on random-dot stimuli. During the decision formation phase they found high correlations between the strength of the motion signal of the stimulus and activation in the FEF. Importantly, the FEFs are involved in visuo-motor integration relevant for the programming and execution of eye movements [34]; by means of intracranial stimulation within FEF saccades can be elicited [48]. Furthermore the FEFs are associated with the allocation of visuo-spatial attention, a process that is important in the majority of video games.

Although our approach to explore the structural basis of video gaming was purely exploratory in nature, the fact that we found higher cortical thickness jointly in left DLPFC and left FEF could suggest that these morphometric correlates are related to training effects in perceptual decision-making and allocation of attention. Perceptual decision-making involves different processes, namely the intake and accumulation of sensory evidence, subsequent categorization and decision-making based on this input and subsequent actions [49]. Video gaming may likewise engage cognitive processes related to perceptual decision-making. In most games, visual stimuli on the screen need to be processed in order to decide which action is required to reach the overall goal. Although the demonstrated association between hours of weekly video game playing with cortical thickness in left DLPFC and left FEF does not allow the conclusion that these morphometric alterations are caused by video game playing and in particular perceptual decision-making within the latter, there is behavioural evidence that implies causality. A study by Green and colleagues [3] has demonstrated more efficient use of sensory evidence in participants with extensive action video game experience. Importantly, action video game training (50 hours) led to qualitatively similar results in a group of participants that did not habitually play action video games in their spare time. These improvements were not restricted to the visual modality, but appeared in the auditory modality as well. The findings by Green and colleagues establish a causal relationship between action video game experience and improved probabilistic inference. Using a neural network, they successfully simulated the improved performance of frequent video game players by means of enhancing the connection strength between the layer providing sensory evidence that would be localized in lower perceptual brain regions (e.g. V5/MT (middle temporal) in a motion discrimination tasks) and the layer that integrates evidence that could be located in higher-level brain regions such as left DLPFC or FEFs. The fact that video gamers perform better in perceptual decision-making is appealing because it provides a mechanism to explain why video game training improves participants' performance in such seemingly different tasks as contrast detection, visual search, multiple object tracking, letter recognition with flankers, and decision-making [4], [5], [7], [50]. However, such an improvement may have wider implications; it could be that frequent video game players perform better because they learn a better model of the stimuli and therefore decide more accurately (Law and Gold 2008). This would be in line with theories of perceptual learning proposing that learning occurs by means of template matching via reweighting the connections between the stages of sensory processing and decision-making [51], [52]. Improvements of probabilistic inference may explain the broad transfer effects resulting from training with video games, e.g. [11], [53] in comparison to the majority of classical cognitive training studies showing only limited transfer with other, even closely related tasks [54], [55].

The presented findings complement previous VBM results on a similar sample in which we have shown that frequent (>9 h/week) compared with infrequent (<9 h/week) video gamers have higher grey matter volume in the left ventral striatum [22]. Furthermore we found higher reward task-related brain activity during feedback of loss compared with feedback of no loss in frequent video gamers. Furthermore we found a negative correlation between deliberation time in a betting task and grey matter volume in the ventral striatum. Taken together results were interpreted in the light of alterations in the reward system and a potential neural correlate for gambling-related decision making. The cortical thickness finding presented in the present analysis broadens this perspective and extends the observed neural correlates to brain regions associated with perceptual decision-making.

Moreover, the observed results fit well to our recent finding of training-related grey matter volume increases in the right DLPFC [56]. A sample of video game-naïve subjects was asked to play the platformer game Super Mario 64 30 min/day for a period of two month. VBM comparing grey matter before and after this intervention revealed significant grey matter growth in right DLPFC, right hippocampus and the cerebellum. Why the observed training effects are right-lateralized, whereas the structural correlates of video gaming hours per week are left-lateralized needs further exploration. Potentially the different lateralization may be due to the fact that the participants in the present study did not only play games such as the platformer we used in the training study.

The fact that we found a significant difference of DLPFC cortical thickness between gamers classified as excessive or addicted gamers based on the CSV-S score, argues in favour of an effect that sets in once a certain intensity of video gaming is reached. For studies aiming to use video games as a tool for training, the present study may be seen as an indication that a considerable amount of training is needed to potentially detect structural effects, provided that the effects observed are a cause of the video gaming. Interestingly, previous research on online gaming addiction in an adolescent sample has shown decreases of cortical thickness in lateral orbitofrontal cortex among other regions [57]. More research is needed to explore the different degrees of addiction to video games.

Future research may focus on the direct assessment of the relationship between cortical thickness in left DLPFC and FEF and perceptual decision-making as well as its association with the different video game genres such as action video games, strategy games, platformers etc., Moreover it would have been interesting to know which game genres the adolescents played most, since in particular action video games have been associated with improvements in cognitive performance; therefore future studies should carefully screen how many hours per week are devoted to which type of video game. Likewise, longitudinal studies should be conducted to elucidate the causal effects of video gaming on cortical thickness in DLPFC and FEF. Furthermore, it would be interesting to assess structural correlates of cumulative video gaming hours over the lifetime instead of current video game frequency and the additional assessment of physical activity and BMI.
